# 2′-fucosyllactose alone or combined with resistant starch increases circulating short-chain fatty acids in lean men and men with prediabetes and obesity

**DOI:** 10.3389/fnut.2023.1200645

**Published:** 2023-07-17

**Authors:** Emanuel E. Canfora, Lars M. M. Vliex, Taojun Wang, Arjen Nauta, Freek G. Bouwman, Jens J. Holst, Koen Venema, Erwin G. Zoetendal, Ellen E. Blaak

**Affiliations:** ^1^Human Biology, School for Nutrition and Translational Research in Metabolism (NUTRIM), Maastricht University Medical Center+, Maastricht, Netherlands; ^2^Laboratory of Microbiology, Wageningen University and Research, Wageningen, Netherlands; ^3^FrieslandCampina, Amersfoort, Netherlands; ^4^NovoNordisk Center for Basic Metabolic Research and Department of Biomedical Sciences, Faculty of Health and Medical Sciences University of Copenhagen, Copenhagen, Denmark; ^5^Maastricht University—Campus Venlo, Centre for Healthy Eating and Food Innovation, Venlo, Netherlands

**Keywords:** short-chain fatty acids, human milk oligosaccharides, obesity, prediabetes, substrate metabolism

## Abstract

**Background:**

Infusion of short-chain fatty acids (SCFA) to the distal colon beneficially affects human substrate and energy metabolism. Here, we hypothesized that the combination of 2′-fucosyllactose (2′-FL) with resistant starch (RS) increases distal colonic SCFA production and improves metabolic parameters.

**Methods:**

In this randomized, crossover study, 10 lean (BMI 20–24.9 kg/m^2^) and nine men with prediabetes and overweight/obesity (BMI 25–35 kg/m^2^) were supplemented with either 2′-FL, 2′-FL+RS, or placebo one day before a clinical investigation day (CID). During the CID, blood samples were collected after a overnight fast and after intake of a liquid high-fat mixed meal to determine plasma SCFA (primary outcomes). Secondary outcomes were fasting and postprandial plasma insulin, glucose, free fatty acid (FFA), glucagon-like peptide-1, and peptide YY concentrations. In addition, fecal SCFA and microbiota composition, energy expenditure and substrate oxidation (indirect calorimetry), and breath hydrogen excretion were determined.

**Results:**

In lean men, supplementation with 2′-FL increased postprandial plasma acetate (*P* = 0.017) and fasting H_2_ excretion (*P* = 0.041) compared to placebo. Postprandial plasma butyrate concentration increased after 2′-FL and 2′-FL+RS as compared to placebo (P < 0.05) in lean men and men with prediabetes and overweight/obesity. Additionally, 2′-FL+RS decreased fasting and postprandial plasma FFA concentrations compared to placebo (P < 0.05) in lean men.

**Conclusion:**

Supplementation of 2′-FL with/without RS the day before investigation increased systemic butyrate concentrations in lean men as well as in men with prediabetes and obesity, while acetate only increased in lean men. The combination of 2′-FL with RS showed a putatively beneficial metabolic effect by lowering plasma FFA in lean men, indicating a phenotype-specific effect.

**Clinical trial registration:**

nr. NCT04795804.

## 1. Introduction

The gut microbiota and its metabolites affect host substrate and energy metabolism (1–4) By the fermentation of indigestible foods, e.g., dietary fibers, the gut microbiota produces important metabolites such as the short-chain fatty acids (SCFA) acetate, butyrate, and propionate (5, 6). A body of rodent studies demonstrated that SCFA administration via different routes prevents body weight gain and improves glucose homeostasis (7–15). Thus, SCFA might be essential regulators in the crosstalk between the gut microbiota and host energy and substrate metabolism.

Human studies support the beneficial effect of gut-derived SCFA on host metabolism and suggest that the site of colonic SCFA production is of importance. Two randomized clinical trials demonstrated that acute infusions of acetate (16) and SCFA mixtures (17) into the distal colon of normoglycemic, overweight men increased circulating acetate concentrations, energy expenditure, lipid oxidation, and circulating levels of peptide YY (PYY), and attenuated whole-body lipolysis. Interestingly, after the distal colonic infusions of SCFA mixtures, increments in circulating acetate correlated positively with increments in lipid oxidation and energy expenditure (17). In contrast, no effects on circulating acetate concentrations and metabolic parameters were observed when acetate was administered in the proximal colon (16).

A potential strategy to increase the SCFA availability in the distal colon is to combine fermentable carbohydrates that differ in degree of polymerization and side chain configuration. In pigs, the use of resistant starch (as more rapidly, but steadily fermentable fiber) shifted more complex carbohydrates to be fermented in the more distal colonic site (18). In a recent study (19), 1-day supplementations of resistant starch with long-chain inulin increased distal colonic fermentation (as shown by increased circulating butyrate and breath hydrogen the next morning), which was coincident with increased energy expenditure, circulating PYY, and improved insulin sensitivity in lean men, but not in men with prediabetes and overweight/obesity. This study and others (20–23) suggest that the capacity of the gut microbiome to yield SCFA as well as the metabolic benefits of an enhanced SCFA availability differs between healthy individuals and metabolically compromised individuals.

In the past years, the interest in human milk oligosaccharides (HMOs), which are a family of unconjugated glycans present in high concentrations in human milk of secretor-positive mothers, has increased. 2′-fucosyllactose (2′-FL) is one of the most prevalent HMOs, making up about 30% of all HMOs in breast milk (24). The majority of ingested 2′-FL reaches the colon where it acts as a selective substrate for specific gut bacteria and is considered a prebiotic (25, 26). Studies in animals showed that oral 2′-FL intake has a beneficial effect on the gut microbiota composition (i.e., increase in Rumminococci and Bifidobacteria), immunity and control of energy intake, fat mass, and body weight (27). In an *in vitro* model of the human gut using the fecal microbiota of infants, 2′-FL was fermented along the entire colon and an increased SCFA production was observed in the distal colon (28).

In the present study, we hypothesized that a combination of 2′-FL and resistant starch (RS) will enhance microbial SCFA production in the distal colon, consequently leading to beneficial effects on host substrate and energy metabolism. Since gut microbial fermentation capacity and SCFA metabolism may differ between metabolic phenotypes, both lean and individuals with overweight/obesity and prediabetes were included in this study.

First, we investigated the potential of 2′-FL alone and 2′-FL in combination with RS on SCFA production in the distal colon in a validated *in vitro* model of the colonic milieu using the fecal microbiota of lean adults and of adults with prediabetes and overweight/obesity. Subsequently, in a crossover study, the metabolic effects of 1-day supplementation of 2′-FL+RS vs. 2′-FL alone or placebo in lean, normoglycemic men as well as men with overweight/obesity and prediabetes were studied. In the morning after 1 day of supplement intake, fasting and postprandial circulating SCFA concentrations (primary outcomes), breath hydrogen, substrate oxidation and energy expenditure, plasma metabolites and hormones, as well as fecal microbiota composition and SCFA concentrations were determined.

## 2. Methods

### 2.1. Participants

Ten normoglycemic lean men and 12 men with overweight/obesity and prediabetes aged between 30 and 65 years were recruited in the Netherlands between February 2020 and October 2021. The study recruitment was stopped due to the part-time closure of the Metabolic Research Unit Maastricht related to COVID-19 from March to July 2020. Screening was performed to assess, via anthropometry measures (height, body weight, waist, and hip circumference), an oral glucose tolerance test (OGTT), and medical history and general health questionnaires, whether the volunteers are eligible to participate. Inclusion criteria for the lean men group were a BMI between 20 kg/m^2^ and 24.9 kg/m^2^, fasting plasma glucose <6.1 mmol/L, and plasma glucose 2 h after the start of the OGTT <7.8 mmol/L. For the group of men with overweight/obesity and prediabetes, men with a BMI between 25 kg/m^2^ and 34.9 kg/m^2^, impaired fasting glucose (plasma glucose ≥6.1 mmol/L and ≤7.0 mmol/L), and/or impaired glucose tolerance (plasma glucose ≥7.8 mmol/L and ≤11.0 mmol/L) were included. The volunteers were Caucasian, with a systolic blood pressure of 100–140 mmHg and diastolic blood pressure of 60–90 mmHg, and indicated that they were weight was stable for at least 3 months (±2 kg) before the screening. Exclusion criteria included the diagnosis of T2D, gastroenterological complaints/diseases, prior abdominal surgery, cardiovascular diseases, and liver or kidney malfunction. In addition, men with a life expectancy shorter than 5 years, or following a hypocaloric diet, using antibiotics and pre- or probiotics in the 3 months before the start of the study were excluded. The included subjects did not use β-blockers, glucose or lipid-lowering medication, or corticosteroids. This human study was approved by the Medical Ethical Committee of Maastricht University Medical Center+ and was conducted in accordance with the Declaration of Helsinki (revised version, October 2008, Seoul, South Korea). Monitoring was performed by the independent Clinical Trial Center Maastricht, Maastricht, The Netherlands. Written informed consent was obtained from all volunteers. All authors had access to the data of the clinical trial and reviewed the final version of the manuscript.

### 2.2. Study design

First, potential distal colonic SCFA production from 2′-FL alone and 2′-FL in combination with resistant starch were assessed using a validated *in vitro* model of the colon (29). Here, pooled fecal microbes that were sampled anaerobically from lean, normoglycemic adults (*n* = 11, aged 30–65 years, BMI ≥20 kg/m^2^ and ≤ 24.9 kg/m^2^, fasting plasma glucose <6.1 mmol/L) and from adults with prediabetes and overweight/obesity (*n* = 14, aged 30–65 years, BMI ≥30 kg/m^2^ and ≤ 40 kg/m^2^, fasting plasma glucose ≥6.1 mmol/L) were used, as previously described (19, 29). In brief, by increasing the pH over a 24-h period (pH 5.8–7.0), the conditions in the colonic proximal region, the colon transversum, and the distal colonic part were simulated. Thereby, the transit of the carbohydrates and their microbial fermentation throughout the whole colon were mimicked, where the last 16 h simulated the processes at the distal colonic site. Samples were taken 1, 2, 4, 6, 8, and 24 h after the addition of 7.5 g of 2′-FL (FrieslandCampina Ingredients, The Netherlands) alone or in combination with 7.5 g of resistant starch (RS2 tapioca starch, Avebe, Veendam, The Netherlands) for SCFA analysis.

Subsequently, in the crossover-designed clinical trial, participants were allocated to the intake of 2′-FL alone and 2′-FL in combination with resistant starch or placebo in random order. An independent researcher randomly assigned participants, using a computer-generated randomization plan, to the order in which they receive the supplements. This was a double-blind study; therefore, the order of the intervention was blinded for both the researcher as well as for the subjects. The content and packaging of the indigestible carbohydrate products and control sachets looked identical. All intervention products were supplemented one day before a clinical investigational day (CID) with a washout period of at least 14 days between CID. The volunteers were asked to fill in a 3-day dietary record directly for the days before each CID. The food diaries were assessed and analyzed using the Dutch food composition database (National Institute for Public Health and Environment, Ministry of Health and Welfare and Sport, The Hague, The Netherlands).

One day before each CID, the subjects refrained from food products rich in indigestible carbohydrates by providing them with alternative foods low in indigestible carbohydrates (e.g., white bread instead of whole-grain bread). On the same day, they also supplemented the carbohydrate (mixture) or the control (see interventions). In addition, the volunteers refrained from alcohol and intensive physical activity for 2 days before each CID.

### 2.3. Interventions

In this crossover-designed randomized clinical trial; the participants consumed three isocaloric supplements. the intervention was the 1-day consumption of either 2′-FL with maltodextrin (2′-FL), 2′-FL with resistant starch (2′-FL+RS), or a maltodextrin placebo, in identical caloric amounts (181.6 kj/d) before each CID. the three supplements were: 1. 2′-FL: 12 g (3 x 4 g) 2′-fucosyllactose (frieslandcampina ingredients, the netherlands) in combination with 5.43 g (3 x 1.81 g) maltodextrin (glucidex IT 12, roquette freres, lestrem, france) to make it isocaloric to the other interventions. 2. 2′-FL+RS: 12 g (3 x 4 g) of 2′-FL in combination with 9.39 g [3 x 3.13 g (80% resistant starch RS2 (3 x 2.5g)] granular potato starch (avebe, veendam, the netherlands). 3. PLA: 11.43 g (3 x 3.81 g) maltodextrin.

The carbohydrate (mixture) and placebo were provided to the subjects in sachets. All intervention products were packed at the food-grade kitchen of the Metabolic Research Unit Maastricht, The Netherlands. All products were mixed in yogurt (0.86 MJ). The volunteers consumed the supplements with their breakfast, lunch, and dinner. A dinner was provided to standardize their evening meal. The standardized dinner provided 1.7 MJ, consisting of 62E% carbohydrates, 24E% protein, and 14E% fat. The subjects consumed the last supplement with the standardized dinner ~14 h before the CID started the next morning.

### 2.4. Clinical investigation days

The day after the intake of the intervention products or placebo, the participants came fasted (>12 h) to the Metabolic Research Unit Maastricht for the CID. After insertion of a cannula into the antecubital vein, substrate oxidation and energy expenditure (indirect calorimetry) and breath H_2_ excretion were measured and blood was sampled during fasting and for 4 h after a liquid high-fat mixed meal. The liquid high-fat mixed meal provided 2.6 MJ, consisting of 61E% fat (35.5E% saturated fat, 18.8E% monounsaturated fat, and 1.7E% polyunsaturated fat), 33E% carbohydrates, and 6E% protein. The liquid meal consists of 125 g whole milk “Campina volle melk” (FrieslandCampina, The Netherlands), 50 g whipped cream “Campina slagroom” (FrieslandCampina, The Netherlands), 150 g whipped ice cream “Hertog slagroomijs” (Unilever, The Netherlands), and 15 g sugar (Royal Consun, The Netherlands). At each CID, indirect calorimetry measures were performed using an open-circuit ventilated hood system (Omnical, Maastricht University, The Netherlands). VCO_2_ (L/min) and VO_2_ (L/min) were determined during fasting and at 30, 60, 120, 180, and 240 min after the meal. The equations of Weir and Frayn (30, 31) were used to assess energy expenditure, lipid, and carbohydrate oxidation. With a hand-held breath analyzer (Bedfont EC60 Gastrolyzer, Rochester, UK), breath H_2_ excretion was assessed during fasting and at 30, 60, 90, 120, 150, 180, and 240 min after meal intake. In addition, the participants collected feces in the morning before each CID.

#### 2.4.1. Biochemical analyses

During the CID, blood was sampled into appropriate pre-chilled tubes before and 30, 60, 120, 180, and 240 min after the meal. The samples were centrifuged at 3,000 g, at 4°C for 15 min, plasma was aliquoted and directly snap-frozen in liquid nitrogen and stored at −80°C until analysis. To determine plasma FFA and glucose, blood was sampled in EDTA tubes (Sigma, Dorset, UK) and an enzymatic assay on an automated spectrophotometer (ABX Pentra 400 autoanalyzer, Horiba ABX, Montpellier, France) was used. Plasma insulin was assessed by the use of a singleplex assay of MesoScale Discovery (Meso Scale Discovery, Gaithersburg, MD 20877 USA). Meso Scales provides a black 96-well plate with capture antibodies against insulin patterned on a distinct spot in each well. Approximately 25 μl samples per well, detection antibodies, and read buffer for electrochemiluminescence were applied according to the instructions of the producer. Plates were read using a SECTOR® Imager. The detection ranges of the assay for insulin is 7.5–50,000 pg/mL. Insulin values were converted to pmol/l using a molar mass of 5,808 g for insulin. A 2-mL EDTA tube containing 20 μL of dipeptidyl peptidase-IV inhibitor (Millipore, Darmstadt, Germany) was sampled for GLP-1 analysis. Plasma samples were assayed for total GLP-1 immunoreactivity using an antiserum, which reacts equally with intact GLP-1 and the primary (N-terminally truncated) metabolite as previously described (32). A 2-mL aprotinin tube containing 20 μL of dipeptidyl peptidase-IV inhibitor was used to sample blood for plasma PYY analysis. Total PYY was assessed as described previously (33) using a monoclonal antibody MAB8500 (Abnova, clone RPY-B12), which reacts equally well with PYY_1 − 36_ and PYY_3 − 36_. For plasma SCFA, blood was collected in a 4-mL Lithium Heparin tube (BD, Plymouth, UK) and analyzed before and 60, 120, and 240 min after the meal. Plasma acetate, propionate, and butyrate concentrations were determined using liquid chromatography–mass spectrometry (LC-MS) as previously described (34).

#### 2.4.2. Fecal SCFA concentration and microbiota composition

On the morning of each CID, the participants collected feces and distributed them to different tubes for SCFA and microbial composition analysis. The fecal samples were transported using ice packs and immediately stored at −80°C upon arrival at Metabolic Research Unit Maastricht. Fecal SCFA was measured using ion exchange chromatography with conductivity detection (Brightlabs, Venlo, The Netherlands) as described before (35) and normalized to dry weight.

For the analysis of fecal microbiota composition, DNA was isolated from 0.25 g feces with repeated bead beating followed by automated isolation and purification using a Maxwell 16 Tissue LEV Total RNA Purification Kit (Promega, Madison, U.S.). The V4 region of the 16S rRNA gene was amplified with uniquely barcoded primers 515F (5′-GTGCCAGCMGCCGCGGTAA)−806R (5′-GGACTACHVGGGTWTCTAAT) (36) Every sample was amplified in triplicate using Phusion hot start II high fidelity polymerase (Thermo Scientific, Waltham, U.S.) with the following cycling conditions; reactions were held at 98°C for 30 s with amplification proceeding for 25 cycles at 98°C for 10 s, 50°C for 10 s, 72°C for 10 s, and a final extension of 7 min at 72°C as previously described (37) PCR products were checked for correct size on a 1% agarose gel and thereafter combined and purified with magnetic beads using the CleanPCR kit (CleanNA, Alphen aan den Rijn, The Netherlands). Thereafter, purified PCR products were quantified with Qubit using the dsDNA BR Assay Kit (Invitrogen, California, USA) and a combined sample for sequencing was created by combining equimolar amounts of amplicons (200 ng) from the individual samples. The resulting libraries were sent to Novogene Co., Ltd. for 2X150nt sequencing on an Illumina Novaseq 6000 instrument. Raw sequencing data were processed using NG-Tax 2.0 with default settings (38) for Amplicon Sequence Variant (ASV) picking and for taxonomic assignments using the SILVA database (version 132) (39) Microbial sequencing data were submitted to the European Nucleotide Archive database under accession number PRJEB57807.

### 2.5. Sample size calculation and statistical analysis

Based on a previous clinical trial of our laboratory (17), also using a randomized, crossover design and a comparable statistical model for analysis, it was estimated that an increase of 30% (with an SD of 5%) in circulating acetate concentrations would be considered a physiologically relevant metabolic effect. Samples size was calculated with Gpower (Version 3.1 for Mac, Parkville, Victoria, Australia), and indicated that nine subjects per group (lean men group and group with men with overweight/obesity and prediabetes) would be sufficient to detect the estimated difference between interventions with a power of 80% at an alpha level of *P* = 0.05. Considering a putative dropout rate of 20%, ten lean and twelve individuals with overweight/obesity and prediabetes were recruited.

Baseline characteristics of lean men and men with overweight/obesity and prediabetes were analyzed for differences by a Student's independent-samples *t*-test. Postprandial responses in indirect calorimetry, metabolic plasma markers, and breath H_2_ excretion were expressed as total area under the curve (AUC0-240) or divided into periods of 2 h (AUC0-120 and AUC 120-240). Differences in fasting (t0) and postprandial AUC between the three treatments were analyzed using a linear mixed model for repeated measures. Treatment was set as a fixed factor and participants were set as a random factor. Although no carry-over effects were expected due to the 14-day washout period, a putative carry-over effect was tested by adding “period” (order of treatments) as a fixed factor. A carry-over effect was not present in the assessed parameters. *Post-hoc* comparison of treatments was performed when the overall treatment effect (Type III Test of Fixed Effects) was at least *P* < 0.1. Due to the explorative nature of the trial, the least significant testing was performed for *post-hoc* comparison. In-text described *P*-values show *post-hoc* comparison of *P*-values. The *P*-values for the overall treatment effect are indicated in the legends of the corresponding figure. The described statistics were performed using SPSS 28.0, and *P* < 0.05 (two-sided *P*-value) was considered statistically significant.

For microbiota composition, all analyses were performed in R version 3.4.0 (40) We calculated alpha diversity and beta diversity at the ASV level using *phyloseq* *(**41**)*. ASV richness and Shannon diversity for alpha diversity were used in this study. Principal Coordinate Analysis (PCoA) based on weighted UniFrac distance and unweighted UniFrac distance was performed to visualize beta diversity. Weighted UniFrac distance takes both the presence and abundance of each ASV into account, while unweighted UniFrac provides equal weight to all ASVs, thereby focusing on the presence or absence of low-abundance ASVs (42). Permutational Multivariate Analysis of Variance was determined to assess whether the intervention was significantly associated with microbiota variation. Within one participant, UniFrac distances were calculated to assess the effect sizes of both intervention treatments on the microbiota compared to the placebo. To determine whether there were pairwise differences in diversity between groups, a Wilcoxon test was performed with Benjamini–Hochberg false-discovery rate for the correction for multiple testing. A *P*-value (or corrected) of <0.05 was considered significant.

## 3. Results

### 3.1. *In vitro* pre-screening

In a validated *in vitro* model of the human colon (29), the addition of RS to 2′-FL resulted in an increased cumulative acetate, butyrate, and total SCFA production for the last 16 h (simulating production in the distal colon) vs. 2′-FL alone (Supplementary Figure 1) using the fecal microbiota pool of lean donors. Over a 24-h period, overall cumulative acetate, butyrate, and total SCFA accumulation were higher with 2′-FL+RS vs. 2′-FL alone when fecal microbiota of individuals with overweight/obesity and prediabetes was used; however, the SCFA production during the last 16 h was not increased.

### 3.2. Randomized clinical trial

In total, 28 men were invited for a physical screening in the Metabolic Research Unit Maastricht, Maastricht University Medical Center+, The Netherlands. Ten normoglycemic lean men and 12 men with overweight/obesity and prediabetes met the criteria for inclusion and were randomized. One participant in the pre-diabetic group stopped after the first CID, due to the COVID-19 measures. Two men in the pre-diabetic group did not continue the study after the first or the second CID due to personal circumstances. The lean men and men with overweight/obesity and prediabetes were comparable in age and height but differed in weight, BMI, waist and hip circumference and waist-hip-ratio, glucose concentrations at fasting and 2 h after the glucose load and glycated hemoglobin (HbA1c) (Table 1).

**Table 1:** Study participants' baseline characteristics (*n* = 19).

**Variables**	**Lean (*****n*** = **10)**		**Pre-diabetic (*****n*** = **9)**		***P*-value**
	**Mean**	**SD**	**Mean**	**SD**	
Age, years	58	8	57	9	0.904
Height, m	176.4	6.5	181.2	7.1	0.139
Weight, kg	73.8	7.4	99.6	8.8	<0.001
Body Mass Index, kg/m^2^	23.7	1.3	30.3	2.4	<0.001
Waist (cm)	92.1	7.5	112.0	9.7	<0.001
Hip (cm)	94.9	6.1	106.6	5.0	<0.001
Waist-Hip Ratio	0.97	0.03	1.05	0.05	<0.001
Systolic blood pressure, mmHg	121.9	7.6	129.9	10.3	0.069
Diastolic blood pressure, mmHg	78.8	3.4	85.0	10.6	0.096
Fasting glucose, mmol/L	5.25	0.50	6.06	0.53	0.004
OGTT 2h glucose, mmol/L	4.03	1.07	8.16	2.88	<0.001
HbA1c mmol/mol Hb	33.8	2.0	37.5	3.8	0.017

Values are presented as mean ± standard deviation (SD). Differences between baseline characteristics of lean vs. pre-diabetic participants were analyzed using a Student's independent-samples *t*-test. OGTT, oral glucose tolerance test; Hb1Ac, glycated hemoglobin.

No serious adverse events occurred during the study. Gastrointestinal symptoms after the intake of the carbohydrate (mixtures) or placebo were assessed via a questionnaire. None of the volunteers experienced abdominal cramps, diarrhea, or rumbling. Two lean participants experienced flatulence after 2′-FL intake. One man with prediabetes experienced flatulence and belching on the days of 2′-FL and 2′-FL+RS supplementation. On the morning of the CID, these symptoms were not present anymore. Compliance for supplement intake was 100% as checked by empty sachets returned and intake diaries. Food diaries indicated that there were no differences in the macronutrient intake before the CID (see Supplementary Data 1 and Supplementary Table 1).

#### 3.2.1. Plasma and fecal SCFA concentrations, and breath H_2_ excretion

**Figure 1:**
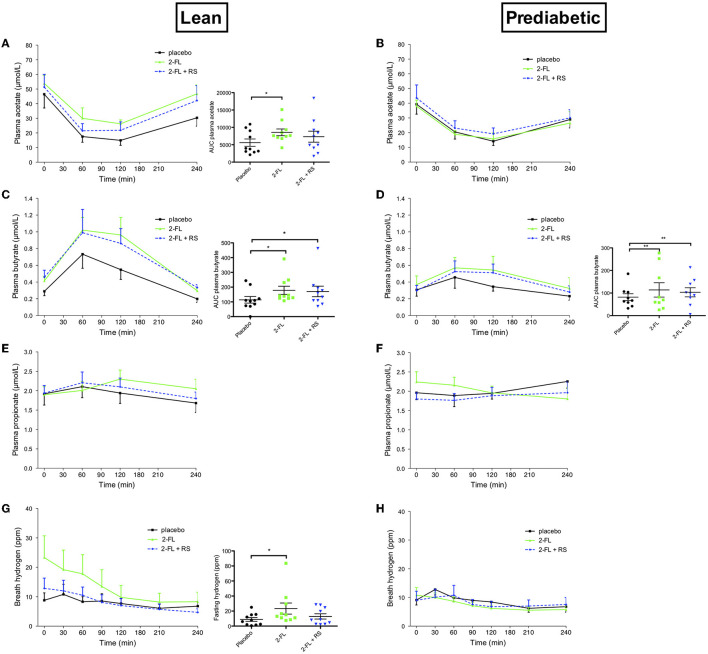
Plasma short-chain fatty acids and breath hydrogen excretion before and after a high-fat mixed meal after providing 2′-FL, 2′-FL+RS, and placebo the day before CID in lean men (lean, *n* = 10) and in men with prediabetes and overweight/obesity (pre-diabetic, *n* = 9). **(A)** Plasma acetate concentrations of lean men. The overall treatment effect for postprandial acetate (AUC0-240) *P* = 0.052. **(B)** Plasma acetate concentrations in pre-diabetic men. **(C)** Plasma butyrate concentrations in lean men. The overall treatment effect for postprandial butyrate (AUC0-240) *P* = 0.023. **(D)** Plasma butyrate concentrations in pre-diabetic men. The overall treatment effect for postprandial butyrate (AUC0-240) *P* = 0.007. **(E)** Plasma propionate concentrations in lean men. **(F)** Plasma propionate concentrations in pre-diabetic men. **(G)** Breath hydrogen excretion of lean men. Overall treatment effect of fasting breath hydrogen *P* = 0.088. **(H)** Breath hydrogen excretion of pre-diabetic men. Values are means ± S.E.M. Differences in fasting (t0) and postprandial AUC between the three interventions (PLA, 2′-FL, and 2′-FL+RS) were analyzed using a linear mixed model for repeated measures and the indicated *P-*values represent *post-hoc* testing. *P < 0.05 vs. placebo, **P < 0.01 vs. placebo, RS, resistant starch; 2′-FL, 2′-fucosyllactose.

**Figure 2:**
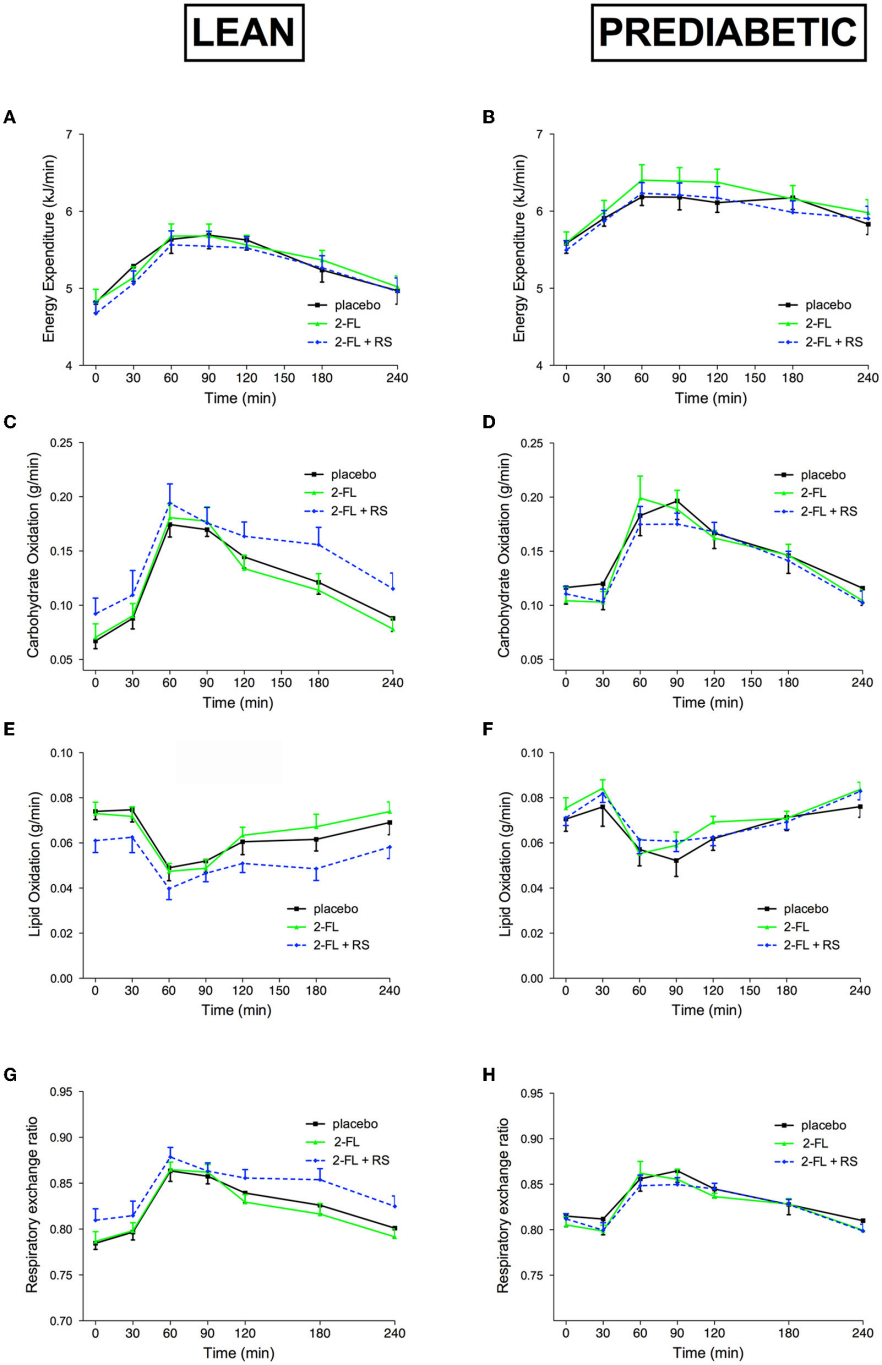
Energy Expenditure and Substrate Oxidation before and after a high-fat mixed meal after providing 2′-FL, 2′-FL+RS, and placebo supplementation the day before CID in lean men (lean, *n* = 10) and in men with prediabetes and overweight/obesity (pre-diabetic, *n* = 9). Energy expenditure of lean **(A)** and pre-diabetic **(B)**, carbohydrate oxidation of lean **(C)** and pre-diabetic **(D)**, lipid oxidization of lean **(E)** and pre-diabetic **(F)**, and respiratory exchange ratio lean **(G)** and pre-diabetic **(H)** men. Values are means ± S.E.M. Differences in fasting (t0) and postprandial AUC between the three interventions (PLA, 2′-FL, and 2′-FL+RS) were analyzed using a linear mixed model for repeated measures. RS, resistant starch; 2′-FL, 2′-fucosyllactose.

In lean, normoglycemic men, fasting plasma acetate concentration did not differ between the three treatments. Postprandial plasma acetate concentration (AUC0-240, *P* = 0.017, Figure 1A) increased after 1-day supplementation of 2′-FL vs. placebo in lean men. Postprandial plasma butyrate concentration increased after 2′-FL (AUC0-240, *P* = 0.011, Figure 1C) and 2′-FL+RS (AUC0-240, *P* = 0.028, Figure 1C) compared to placebo. No difference was observed in fasting and postprandial plasma propionate concentrations. Fasting breath hydrogen excretion increased (*P* = 0.041) with 2′-FL supplementation vs. placebo (Figure 1E).

In men with overweight/obesity and prediabetes, postprandial plasma butyrate concentrations increased after treatment with 2′-FL (AUC0-240, *P* = 0.003, Figure 1D) and 2′-FL+RS (AUC0-240, *P* = 0.009, Figure 1D) compared to placebo. Fasting and postprandial plasma acetate (Figure 1B) and propionate concentrations and breath hydrogen excretion (Figure 1F) did not differ between treatments.

Fecal SCFA concentrations did not differ between treatments in lean men and men with overweight/obesity and prediabetes (Supplementary Figure 2).

#### 3.2.2. Energy expenditure and substrate oxidation

No treatment effects on fasting and postprandial energy expenditure between treatments were found in lean men and men with overweight/obesity and prediabetes (Figures 2A, B, respectively).

In lean men (Figures 2C, E, G) and men with prediabetes and overweight/obesity (Figures 2D, F, H), no differences in carbohydrate and lipid oxidation, as well as respiratory exchange ratio, were observed between the treatments.

#### 3.2.3. Plasma metabolites and hormone concentrations

**Figure 3:**
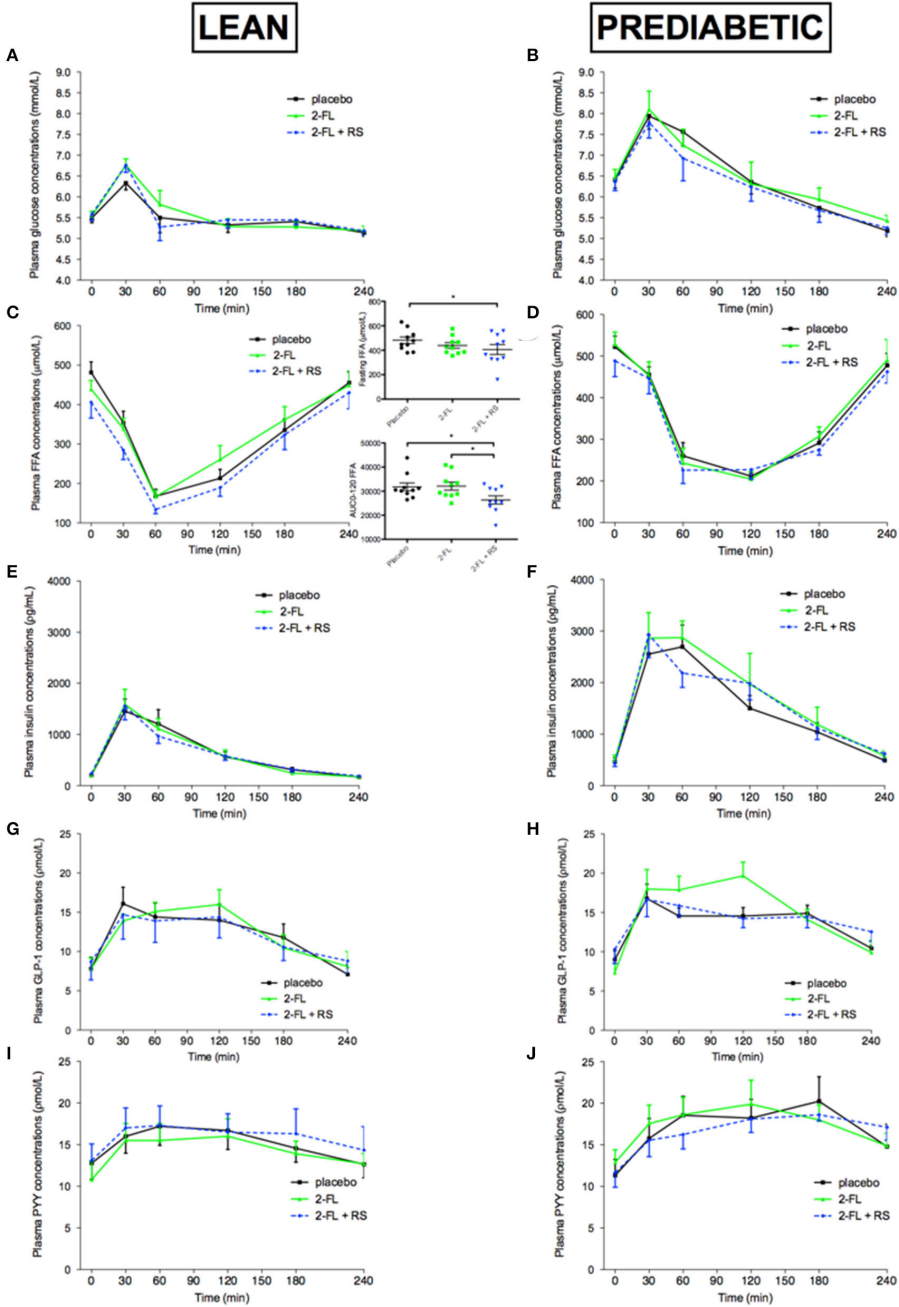
Plasma glucose, FFA, insulin, GLP-1, and PYY concentrations before and after a high-fat mixed meal after providing 2′-FL, 2′-FL+RS, and PLA supplementation the day before CID in lean men (lean, *n* = 10) and in men with prediabetes and overweight/obesity (pre-diabetic, *n* = 9). Plasma glucose concentrations of lean **(A)** and pre-diabetic **(B)**, FFA concentrations of lean [**(C)**, overall treatment effect of fasting FFA *P* = 0.085; and postprandial FFA (AUC0-120) *P* = 0.059] and pre-diabetic **(D)**, insulin concentrations of lean **(E)** and pre-diabetic **(F)**, GLP-1 concentrations of lean **(G)** and pre-diabetic **(H)**, and PYY concentrations of lean **(I)** and pre-diabetic **(J)** men. Values are means ± S.E.M. Differences in fasting (t0) and postprandial AUC between the three interventions (PLA, 2′-FL, and 2′-FL+RS) were analyzed using a linear mixed model for repeated measures and the indicated *P-*values represent *post-hoc* testing. *P < 0.05; RS, resistant starch; 2′-FL, 2′-fucosyllactose; GLP-1, glucagon-like peptide-1; PYY, peptide YY.

In lean men, fasting plasma FFA concentration decreased after treatment with 2FL+RS vs. placebo (*P* = 0.030). In addition, during the first 2 h of the postprandial phase (AUC0-120), plasma FFA concentration decreased after treatment with 2′-FL+RS vs. placebo (*P* = 0.043) and 2′-FL (*P* = 0.033, Figure 3C). Fasting and postprandial plasma glucose, insulin, GLP-1, and PYY concentrations did not differ between intervention groups in lean normoglycemic men (Figure 3).

In men with prediabetes and overweight/obesity, there were no differences in fasting and postprandial plasma FFA, glucose, insulin, GLP-1, and PYY concentrations between treatment groups (Figure 3).

#### 3.2.4. Fecal microbiota profile

**Figure 4:**
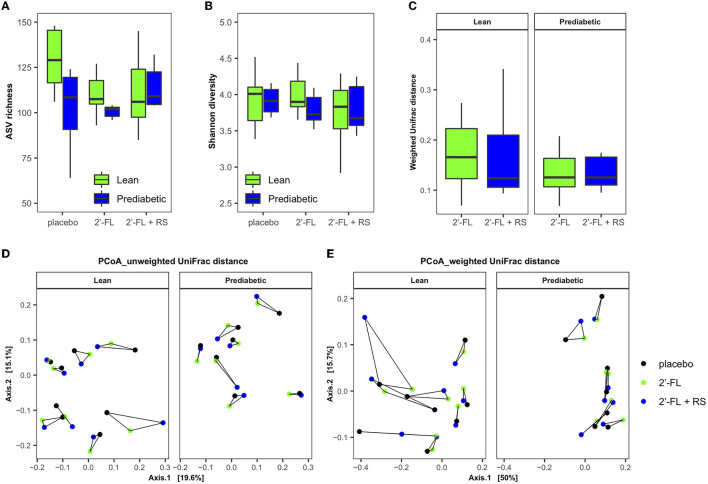
Impact of 2′-FL, 2′-FL+RS, and placebo supplementation on fecal microbiota in lean men (*n* = 8, two individuals were not able to sample enough feces on all 3 days) and men with prediabetes and overweight/obesity (*n* = 7, two individuals were not able to sample enough feces on all day). Fecal microbiota alpha diversity parameters: **(A)** ASV richness and **(B)** Shannon diversity of lean men and men with overweight/obesity and prediabetes. Changes in fecal microbiota composition (weighted and unweighted UniFrac distance): **(C)** Intra-individual microbiota variation compared to placebo after fiber intervention in lean men and men with prediabetes and overweight/obesity. PCoA plots based on **(D)** unweighted and **(E)** weighted UniFrac distance: Samples taken from the same person are connected by solid lines, and the longer the line, the larger the variation. RS, resistant starch; 2′-FL, 2′-fucosyllactose.

Sequencing of the V4 region of the 16S rRNA gene resulted in a mean of 163,346 reads/sample with an SD: ±65,917. The day-after intake of 2′-FL with and without the addition of RS, ASV richness, and Shannon diversity did not differ between treatment groups and did not result in a uniform change in lean men and men with prediabetes and overweight/obesity (Figure 4). Both unweighted and weighted UniFrac-based PCoA showed that the direction of the change in microbiota composition was specific for each individual (Figure 4).

## 4. Discussion

In this double-blind, placebo-controlled, randomized, crossover study, the effects of 1-day intake of 2′-FL or 2′-FL combined with RS on substrate and energy metabolism the morning after intake were studied. In lean men, supplementation of 2′-FL increased postprandial plasma acetate and fasting breath H_2_ excretion compared to placebo. Postprandial plasma butyrate concentration increased after 2′-FL and 2FL+RS as compared to placebo in lean men and men with prediabetes and overweight/obesity. Additionally, 2′-FL+RS decreased fasting and postprandial plasma FFA concentrations compared to placebo in lean men, but not in men with prediabetes and overweight/obesity.

In lean individuals, an increase in microbial fermentation markers (fasting breath hydrogen and postprandial plasma butyrate) after 2′-FL+RS was coincident with decreased fasting and postprandial FFA concentrations without differences in systemic insulin concentrations. These outcomes may suggest that the 2′-FL+RS-induced reduction in circulating FFA levels is insulin independent and is mediated by gut microbial-derived SCFA. In line with these findings, several animal studies demonstrated the beneficial effects of SCFA on host lipid metabolism (4, 9, 14, 43). In addition, an observational study showed that circulating, but not fecal, butyrate concentrations were inversely associated with circulating free fatty acid concentrations in adults with a broad range of BMI and glycemic status (44). Moreover, the administration of SCFA mixtures in the distal colon decreased whole-body lipolysis in men (16). The mechanisms may relate to an SCFA-induced activation of the peroxisome-proliferator activated receptor (PPAR-γ) in adipose tissue, a key regulator in the metabolism of lipids, among others involved in FFA uptake and storage in the adipocyte (45). In addition, *in vitro* studies using white adipocyte models have shown that SCFA, in particular acetate, can inhibit intracellular lipolysis via a G protein-coupled receptor 43 dependent mechanism resulting in partial inhibition of hormone-sensitive lipase phosphorylation (46, 47). Nevertheless, since the intervention with 2′-FL alone also enhanced circulating acetate and butyrate concentrations but did not result in decreased FFA concentrations in lean men, we cannot exclude that there are additional (non-SCFA related) 2′-FL+RS-induced mechanisms involved.

In contrast to the effects in lean men, no metabolic effects were observed after the intake of 2′-FL+RS and 2′-FL+RS-induced increase in circulating butyrate concentrations in men with obesity and prediabetes. In relation to that and in accordance with our findings, a body of clinical trials demonstrated that in metabolically healthy individuals, the response after oral butyrate administration, intravenous acetate, dietary fiber, or vinegar interventions is more effective in improving host substrate metabolism as compared to metabolically compromised individuals (including individuals with metabolic syndrome, hyperinsulinemia, prediabetes, and type 2 diabetes) (20–23, 48). This raises the question whether, although the uptake could be unaffected (downstream), SCFA metabolism and signaling pathways are disrupted in the metabolically compromised phenotype. However, these studies were either acute or short-term interventions and/or used products that do not reach the distal colonic site due to bio-accessibility in the proximal small intestine (orally administered butyrate or acetate) or rapid fermentation in the more proximal colon. In contrast to those studies, a clinical trial showed that a diet rich in complex fibers for 12 weeks decreased HbA1c and fasting blood glucose in individuals with type 2 diabetes (2). Interestingly, when responder analyses of the genomes of the fecal bacteria were performed, the positive responders showed an increased genetic microbial capacity to ferment fibers and produce SCFA, in particular, acetate and butyrate. By contrast, the genome of negative responders harbors genes that are involved in the production of proteolytic metabolites such as indoles and hydrogen sulfide (2). Since proteolytic fermentation mainly occurs in the distal colon, these results suggest that a fiber-induced modification of the microbial functionality (including increased SCFA production in the distal colon) for a longer-term period can translate into improved glucose homeostasis, also in individuals with metabolic disorders.

The potential beneficial reduction in FFA concentrations with 2′-FL+RS in lean individuals seems to be fiber-specific, as these effects were not observed in two clinical trials with different fibers using the same design and metabolic phenotypes (19). In these studies, RS was either combined with long-chain inulin or with yeast beta-glucan. Whereas, in the intervention with yeast beta-glucan, no metabolic effects were observed, the combination of RS with long-chain inulin increased energy expenditure and circulating PYY, and decreased postprandial glucose levels in lean men, but not in men with prediabetes and overweight/obesity. Interestingly, in contrast to 2′-FL, in these two clinical trials, the intake of the fibers alone did not increase circulating SCFA concentrations (19). These findings indicate that 2′FL (despite its relatively low molecular weight) is an interesting resistant, slowly fermentable microbial substrate that potentially reaches the microbiota in the distal colon, which is in line with previously performed *in vitro* fermentation experiments (28).

An important outcome of our clinical trial is that in both phenotypes, a prominent increase in plasma butyrate concentrations was observed after 2′-FL and 2′-FL+RS, which has previously been associated with several beneficial metabolic health benefits (44, 49, 50). This is noteworthy since as demonstrated in a study using isotopically labeled SCFA and dietary fibers (51), only a small proportion (~2%) of butyrate reaches the systemic circulation when produced in the proximal colon. In the proximal region of the colon, the majority of butyrate is directly used by colonocytes as an energy source, or is metabolized in the liver after absorption. In contrast, SCFA produced in the distal region of the colon can partly bypass the liver via rectal veins and thereby drain into the vena cava inferior, reaching the systemic circulation directly (52). In addition, SCFA release from the distal colon to the circulation is higher compared to the proximal colon (53). Both together result in increased SCFA concentrations in the systemic circulation. Thus, the elevated circulating butyrate concentrations suggest that the fermentation of 2′-FL mainly occurred in the distal colon, which is in line with our *in vitro* findings.

This well-controlled crossover study has limitations. Detecting increased or decreased SCFA concentrations in intestinal content or plasma is the net effect of several processes, i.e., production and uptake by the microbiota itself and uptake by the host. Therefore, increased circulating SCFA might be the result of increased microbial fermentation or the effect of endogenous processes contributing to SCFA fluxes. In future experiments, isotopically labeled fibers and/or continuous fermentation kinetics can be used to investigate this. In addition, the subacute design of the study using only male participants can be seen as a limitation as it remains to be determined whether the outcomes can be translated to the whole population and whether the metabolic effects translate into long-term relevance.

In conclusion, the present randomized crossover trial demonstrated that 1-day supplementation of 2′-FL before a clinical investigation day increases acetate and butyrate in the systemic circulation and breath hydrogen concentration in lean men. The addition of RS reduced fasting and postprandial plasma FFA levels in lean men. In men with overweight/obesity and prediabetes, 1-day supplementation of 2′-FL and 2′-FL+RS also increased circulating butyrate concentrations. These outcomes suggest that 2′-FL (alone or in combination with RS) is an interesting nutritional product to target microbial functionality in the distal colon. Therefore, future research should study the effect of long-term supplementation of these microbial substrates and whether it translates into beneficial effects on gut microbiota composition/functionality and metabolic health. Furthermore, the substrate- and phenotype-specific differences should be further investigated using tailored (defining subphenotypes) or even individualized approaches.

## Data availability statement

Sequencing data are deposited in the European Nucleotide Archive. The names of the repository/repositories and accession number(s) can be found below: https://www.ebi.ac.uk/ena, PRJEB57807.

## Ethics statement

The studies involving human participants were reviewed and approved by Medical Ethical Committee of Maastricht University Medical Center+. The patients/participants provided their written informed consent to participate in this study.

## Author contributions

EC conducted the research, acquired data, completed statistical analysis, and wrote the article. LV, TW, FB, JH, and KV generated data. LV, AN, TW, FB, JH, KV, EZ, and EB critically revised the manuscript. EC and EB had primary responsibility for the final content. All authors read and approved the final content.
