# Supplementary material for: 2′-fucosyllactose alone or combined with resistant starch increases circulating short-chain fatty acids in lean men and men with prediabetes and obesity
Source: Front Nutr. 2023 Jul 17;10:1200645. doi: 10.3389/fnut.2023.1200645 (PMC10388544; doi:10.3389/fnut.2023.1200645)
Supplement: Supplementary file 1 [file Data_Sheet_1.docx]

**Supplementary data**

**Supplementary figure 1**. In vitro screening of acetate, butyrate and total SCFA production after supplementation of 2’-FL alone or co-supplementation of 2’-FL and resistant starch. The in vitro model of the human colon was either incubated with microbiota from lean (A, C, E) or from donors with overweight/obesity and prediabetes (B, D, F). Dashed lines (and the values after the +-symbol) indicate (quantitative) changes of SCFA production in the last 16 hours of incubation reflecting the distal colon. RS, resistant starch; SCFA, short-chain fatty acids; 2’-FL, 2’-fucosyllactose

** Supplementary figure 2. Fecal SCFA concentrations after 2’-FL and 2’-FL+RS intake.** Fecal concentrations of acetate of lean (A) and prediabetic (B), propionate of lean (C) and prediabetic (D), and butyrate of lean (E) and prediabetic (F) individuals. For the statistical analysis fecal samples of 9 lean men (one individuals were not able to sample feces on all 3 days) and 8 men with prediabetes and overweight/obesity (one individual was not able to sample feces on all day) men was used. RS, resistant starch; PLA, placebo; 2’-FL, 2’-fucosyllactose

**Food intake**

Three-day food diaries were obtained prior to each CID. One participant of the lean group was excluded from dietary intake analysis due to incomplete documentation of food intake. Energy intake and macronutrient composition on the first two days and third day was not different between intervention groups in lean and prediabetic individuals (supplementary table 1A and 1B, respectively). As instructed, dietary fiber intake on day 3 (day of fiber administration) significantly increased with 2’-FL+RS and 2’-FL compared to placebo in lean and participants with obesity and prediabetes (P < 0.001) and with 2’-FL+RS compared to 2’-FL alone (P < 0.001; Supplementary table 1A and 1B).

| Supplementary Table 1a: THREE-DAY food record. Energy intake and dietary composition for lean men (n =9) | | | | | |
| --- | --- | --- | --- | --- | --- |
| Variable | **Time** | **Treatment** | | | **P-value** |
|  |  | Placebo | 2’-FL | 2’-FL + RS |  |
| Energy intake (kcal) | Day 1+2 (mean) | 2211 ± 594 | 2166 ± 709 | 2299 ± 567 | 0.890 |
|  | Day 3 | 2061 ± 168 | 2123 ± 226 | 2024 ± 329 | 0.738 |
| Carbohydrate intake (g) | Day 1+2 (mean) | 274 ± 83 | 254 ± 95 | 247 ± 49 | 0.340 |
|  | Day 3 | 230 ± 40 | 267 ± 47 | 226 ± 55 | 0.129 |
| Fat intake (g) | Day 1+2 (mean) | 87 ± 29 | 84 ± 31 | 92 ± 37 | 0.380 |
|  | Day 3 | 81 ± 44 | 67 ± 18 | 69 ± 27 | 0.309 |
| Protein intake (g) | Day 1+2 (mean) | 78 ± 21 | 87 ± 33 | 102 ± 52 | 0.296 |
|  | Day 3 | 94 ± 25 | 91 ± 22 | 91 ± 26 | 0.617 |
| Fiber intake (g) | Day 1+2 (mean) | 26 ± 10 | 25 ± 13 | 22 ± 9 | 0.441 |
|  | Day 3 | 16 ± 3 | 27 ± 3 | 35 ± 4 | <0.001* |
| *Values are given as mean ± standard deviation. P-values were obtained by linear mixed model analysis. Significance was set at P < 0.05.* RS, resistant starch; 2’-FL, 2’-fucosyllactose | | | | | |

| Supplementary Table 1b: THREE-DAY food record. Energy intake and dietary composition for men with overweight/obesity and prediabetes (n = 9) | | | | | | |
| --- | --- | --- | --- | --- | --- | --- |
| Variable | **Time** | **Treatment** | | | ***P*-value** | |
|  |  | Placebo | 2’-FL | 2’-FL + RS |  | |
| Energy intake (kcal) | Day 1+2 (mean) | 2300 ± 386 | 2216 ± 440 | 2422 ± 638 | 0.593 |  |
|  | Day 3 | 2297 ± 620 | 2011 ± 561 | 2200 ± 695 | 0.167 |  |
| Carbohydrate intake (g) | Day 1+2 (mean) | 247 ± 82 | 226 ± 75 | 237 ± 70 | 0.735 |  |
|  | Day 3 | 288 ± 122 | 241 ± 76 | 283 ± 144 | 0.351 |  |
| Fat intake (g) | Day 1+2 (mean) | 86 ± 26 | 82 ± 21 | 117 ± 58 | 0.151 |  |
|  | Day 3 | 81 ± 35 | 72 ± 21 | 102 ± 91 | 0.793 |  |
| Protein intake (g) | Day 1+2 (mean) | 106 ± 38 | 92 ± 16 | 107 ± 13 | 0.302 |  |
|  | Day 3 | 104 ± 24 | 101 ± 26 | 105 ± 28 | 0.747 |  |
| Fiber intake (g) | Day 1+2 (mean) | 22 ± 6 | 20 ± 6 | 20 ± 4 | 0.821 |  |
|  | Day 3 | 15 ± 7 | 25 ± 6 | 36 ± 8 | <0.001* |  |
| *Values are given as mean ± standard deviation. P-values were obtained by linear mixed model analysis. Significance was set at P < 0.05*. RS, resistant starch; 2’-FL = 2’-fucosyllactose | | | | | | |
